# Hypertrophic Cardiomyopathy Complicated by Pulmonary Edema in the Postpartum Period

**DOI:** 10.1155/2013/802352

**Published:** 2013-04-03

**Authors:** Kate Hanneman, Elsie T. Nguyen, Andrew M. Crean

**Affiliations:** ^1^Department of Medical Imaging, Toronto General Hospital, University of Toronto, Toronto, ON, Canada; ^2^Division of Cardiology, Peter Munk Cardiac Centre, Toronto General Hospital, University of Toronto, Toronto, ON, Canada

## Abstract

We report the case of a 42-year-old patient with hypertrophic cardiomyopathy (HCM) who presented to the emergency department with severe shortness of breath one week following uneventful cesarean delivery. Thoracic CT ruled out pulmonary embolus and confirmed pulmonary edema. Asymmetric interventricular septal thickening was clearly identified, demonstrating that the heart may be evaluated even on a non-ECG gated study. Acute pulmonary edema in the postpartum period is an unusual clinical presentation of HCM.

## 1. Introduction

Hypertrophic cardiomyopathy (HCM) is an autosomal dominant inheritable condition. Only a few prior studies have reported regarding the course of pregnancy in women with HCM. 

## 2. Case Report

**Figure 1:**
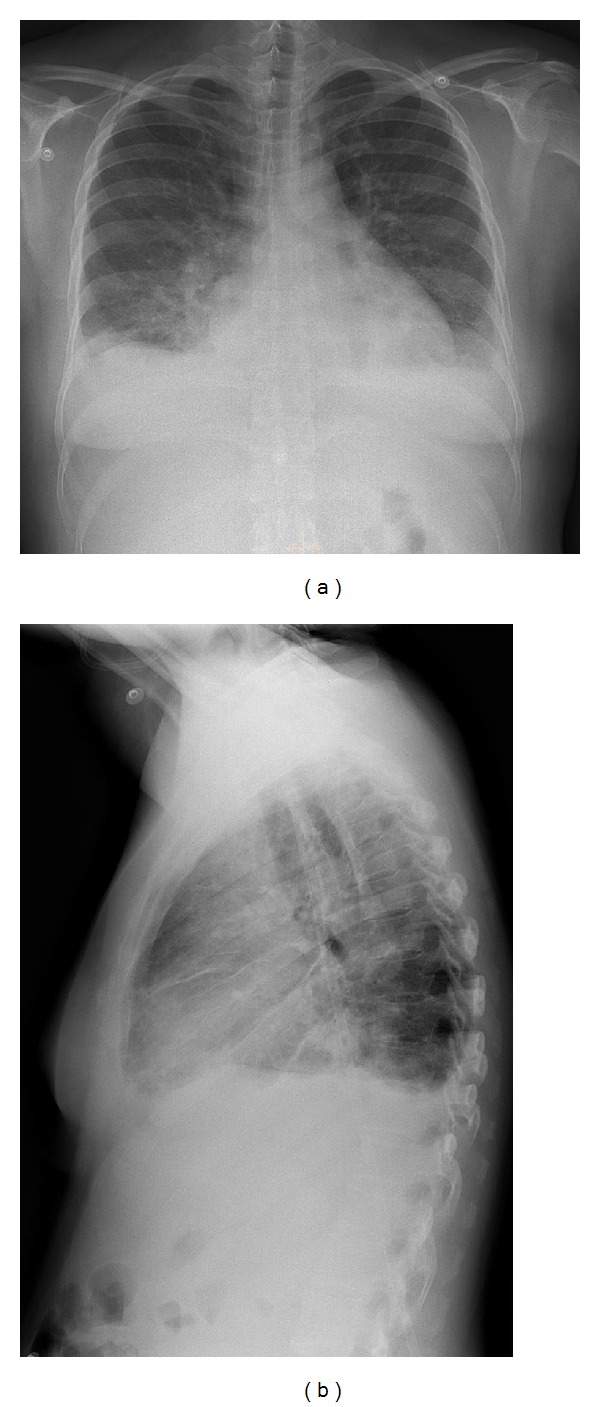
A 42-year-old female with hypertrophic cardiomyopathy and acute pulmonary edema. Findings: chest radiographs (PA (a) and lateral (b)) demonstrate small bilateral pleural effusions, cardiomegaly, and perihilar airspace opacities in keeping with pulmonary edema.

**Figure 2:**
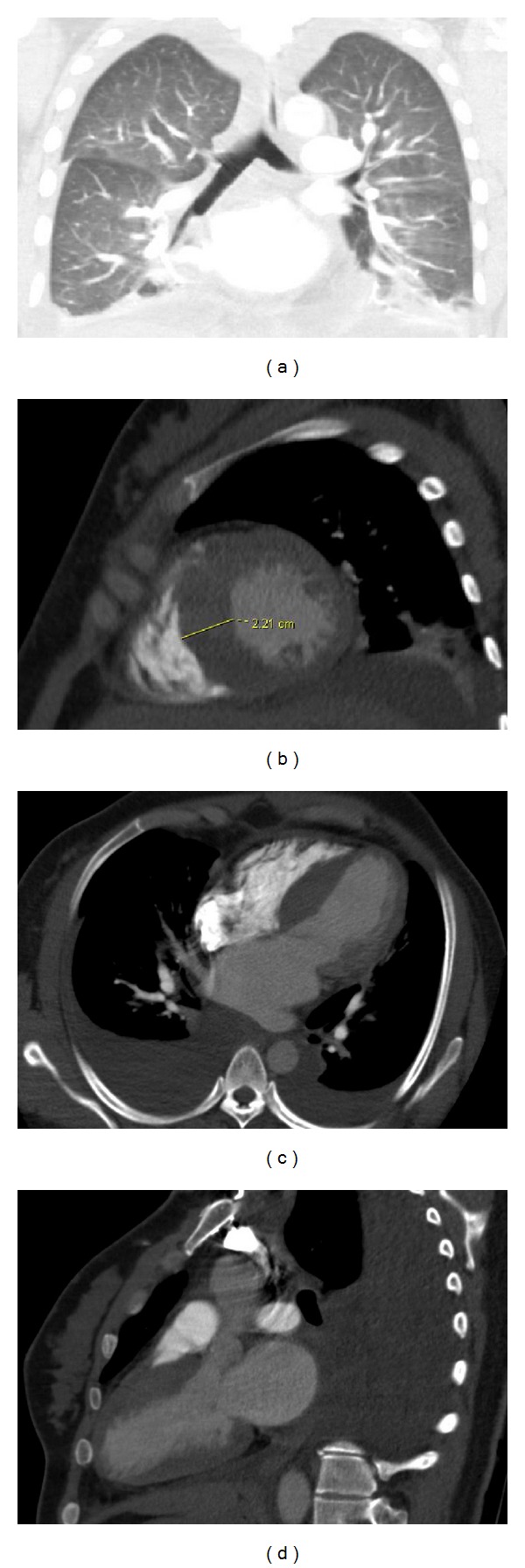
A 42-year-old female with hypertrophic cardiomyopathy and acute pulmonary edema. Findings: CT pulmonary angiogram ruled out pulmonary embolus and demonstrated diffuse, bilateral, and central peribronchovascular ground glass opacities and pleural effusions in keeping with pulmonary edema (coronal image, lung windows, (a)), thickening of the interventricular septum measuring up to 22 mm, and left atrial enlargement (cardiac reconstructions, short axis (b), 4-chamber view (c), and 3-chamber view (d)).

A 42-year-old G4P1 with hypertrophic cardiomyopathy (HCM) presented to the emergency department seven days after uneventful cesarean delivery with severe shortness of breath. Physical exam revealed elevated jugular venous pressure, bilateral leg edema, and bibasilar lung crackles. Chest radiographs demonstrated bilateral pleural effusions and perihilar airspace opacities (Figures 1(a) and 1(b)). Thoracic computed tomography (CT) pulmonary angiography ruled out pulmonary embolus and confirmed pulmonary edema (Figure 2(a)). Asymmetric interventricular septal thickening measuring up to 22 mm was noted, despite the fact that the patient's diagnosis of HCM was not known at the time of reporting (Figures 2(a), 2(b), and 2(c)). 

Subsequent transthoracic echocardiography demonstrated normal systolic function (see videos A and B in Supplementary Material available online at http://dx.doi.org/10.1155/2013/802352). Diastolic parameters were abnormal with an E/E′ of 19 mmHg indicating elevated left atrial pressure and a mild provocable left ventricular outflow tract gradient of 33 mmHg with amyl nitrate. 

The patient was admitted to hospital and improved rapidly following administration of intravenous diuretics. Peripartum intravascular fluid shifts and diastolic impairment likely contributed to heart failure in this patient. 

The patient was discharged home the following day and has remained well with no further complications at followup six months later.

## 3. Discussion

Hypertrophic cardiomyopathy (HCM) is an autosomal dominant inheritable condition with variable penetrance and natural history and is caused by mutations in one of nine genes encoding sarcomeric proteins [1]. The incidence of HCM is approximately 1 in 500 in the general adult population. Myocyte disarray and interstitial fibrosis are the hallmark pathologic features. 

Imaging features are variable given the phenotypic heterogeneity of patients with HCM. Asymmetric septal hypertrophy is the most common variant, accounting for 60–70% of cases [2]. Other patterns include concentric hypertrophy and pure apical involvement. 

The differential diagnosis for myocardial hypertrophy includes restrictive etiologies such as amyloidosis and sarcoidosis, as well as the sequela of systemic arterial hypertension. In most cases of HCM, myocardial hypertrophy and thickening are asymmetric and there is an appropriate clinical and/or family history. Cardiac MRI is particularly useful in differentiating HCM from other causes of myocardial hypertrophy due to the ability of late gadolinium enhanced imaging (LGE) to characterize different patterns of enhancement [2].

Heart disease is present in 0.5–1% of all pregnant women and is the most common cause of death among pregnant women in the developed world [3]. Cardiac output may increase by 30–50% in the first half of pregnancy [4]. Patients with preexisting cardiomyopathies often develop clinical heart failure as the Starling mechanism decompensates in the face of an expanded intravascular volume [4]. The incidence of congestive heart failure in the peripartum period in patients with HCM has been reported at 15–39% and is related to functional status prior to pregnancy [5, 6]. 

Overall, the risk of a cardiovascular event during pregnancy in women with HCM who experience no or mild symptoms before pregnancy is low. However, careful monitoring is recommended particularly in the immediate peripartum period when large fluid shifts can lead to acute pulmonary edema as occurred in this case [4, 7]. In the setting of acute heart failure, therapeutic aims are similar to those in nonpregnant women, and both intravenous diuretics and vasodilator therapy with nitroglycerin can be used safely [4]. 

## 4. Teaching Point

Anatomic and morphologic cardiac information may be gleaned even from a non-ECG gated thoracic CT performed for another indication. Acute pulmonary edema in the postpartum period is an unusual clinical presentation of hypertrophic cardiomyopathy.

## Supplementary Material

Supplementary Material: 42 year old female with hypertrophic cardiomyopathy and acute pulmonary edema. Transthoracic echocardiography (parasternal long axis (Video A) and short axis (Video B)), demonstrate asymmetric septal thickening in keeping with the diagnosis of hypertrophic cardiomyopathy, and normal systolic function. Diastolic parameters were abnormal with an E/E' of 19 mmHg indicating elevated left atrial pressure. There was no resting left ventricular outflow tract (LVOT) gradient. There was a mild provocable LVOT gradient to 33 mmHg with administration of amyl nitrate.
